# Acute retroperitoneal bleeding due to inferior mesenteric artery aneurysm: Case report

**DOI:** 10.1186/1471-230X-10-59

**Published:** 2010-06-09

**Authors:** P Pérez-Vallecillos, R Conde-Muíño, I Segura-Jiménez, N Maldonado-Fernández, JA Ferrón, V García-Róspide, P Palma

**Affiliations:** 1 Department of Vascular Surgery, HUVN, Av. Fuerzas Armadas s/n. Granada, 18014 Granada, Spain; 2 Division of Colorectal Surgery, Department of General Surgery, HUVN. Av. Fuerzas Armadas s/n., 18014 Granada, Spain

## Abstract

**Background:**

Visceral artery aneurysms (VAA), although uncommon, are increasingly being detected. We describe a case of spontaneous retroperitoneal hemorrhage from a ruptured IMA aneurysm associated with stenosis of the superior mesenteric artery (SMA) and celiac trunk, successfully treated with surgery.

**Methods:**

A 65-year-old man presented with abdominal pain and hypovolemic shock. Abdominal CT scan showed an aneurysm of the inferior mesenteric artery with retroperitoneal hematoma. In addition, an obstructive disease of the superior mesenteric artery and celiac axis was observed.

**Results:**

Upon emergency laparotomy a ruptured inferior mesenteric artery aneurysm was detected. The aneurysm was excised and the artery reconstructed by end-to-end anastomosis.

**Conclusions:**

This report discusses the etiology, presentation, diagnosis and case management of inferior mesenteric artery aneurysms.

## Background

Visceral artery aneurysms (VAA), although uncommon, are increasingly being detected. The exact prevalence is not well documented and it is mainly known from autopsies [1,2]. They have a significant potential to rupture and are frequently life-threatening for the patient [3]. The inferior mesenteric artery (IMA) is less affected than other locations such as splenic, hepatic, superior mesenteric and celiac arteries [4]. As most cases are asymptomatic, the real incidence is not known and only isolated cases have been reported. We describe a case of spontaneous retroperitoneal hemorrhage from a ruptured IMA aneurysm associated with stenosis of the superior mesenteric artery (SMA) and celiac trunk, successfully treated with surgery. We also point out the unexpected association between this pathology and the symptoms related.

## Case presentation

**Figure 1:**
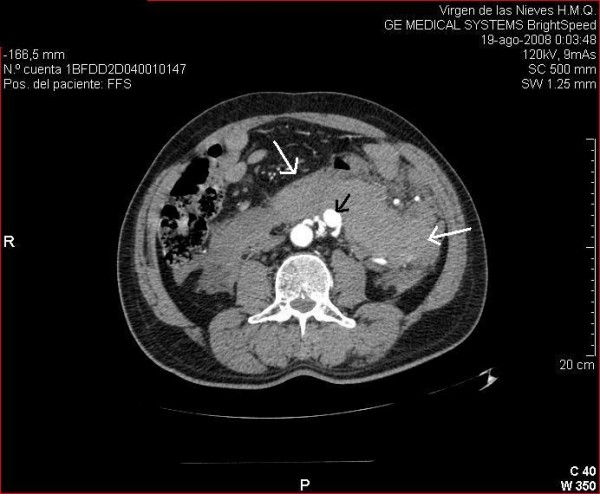
**Retroperitoneal haematoma**. CT image demonstrating a huge retroperitoneal hematoma (white arrows) and the IMA aneurysm (black arrow).

**Figure 2:**
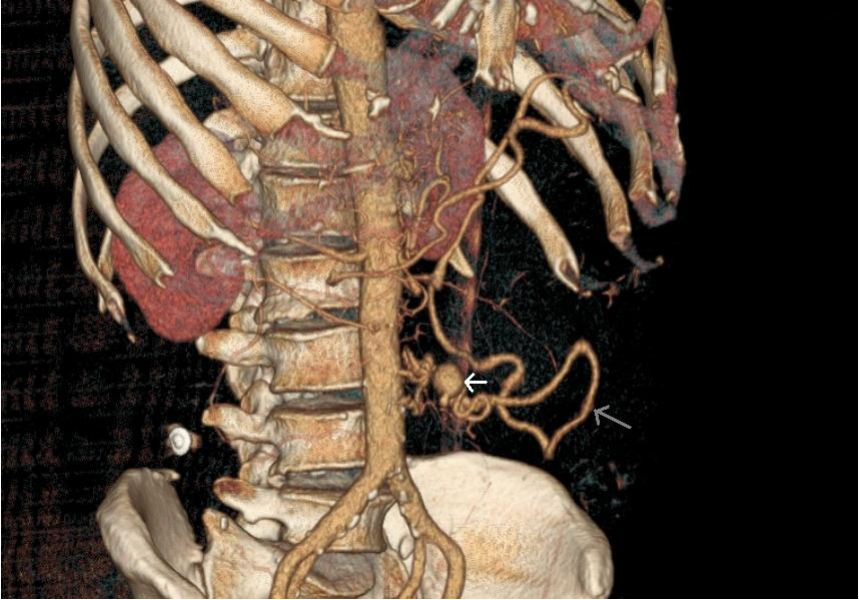
**IMA aneurysm**. CT reconstruction showing the inferior mesenteric artery aneurysm (white arrow), the artery tortuousness and a strongly developed marginal artery (grey arrow).

A 65-year-old man was admitted to hospital with acute abdominal pain and hypovolemic shock. He was a cigarette smoker for more than 20 years, had arterial hypertension and a medical history of cholecystectomy and appendectomy. Abdominal CT imaging showed a huge retroperitoneal hematoma of 7 × 23 × 25 cm (Fig. 1) and an aneurysm of the IMA about 2 cm from its origin resulting in concurrent occlusion of superior mesenteric and celiac arteries (Fig. 2). The IMA also was very wound from its origin. The patient underwent midline laparotomy with a transperitoneal approach to the IMA by a multidisciplinary team of vascular and colorectal surgeons. Severe bowel adherences were observed which possibly helped to limit the massive hemorrhage limited to the retroperitoneum. The aneurysm was completely excised. The normal proximal and distal ends of the IMA were then mobilized. The artery was finally reconnected in an end-to-end manner to re-establish blood flow. Neither a graft nor a shunt was used, with only a short period of ischemia during the running suture. Heparin was rinsed only locally, due to the massive hemorrhage and the need for transfusion. Macroscopic examination of the aneurysm sac demonstrated typical appearances of atherosclerotic aneurismal tissue. There was no history of trauma or any clinical sign of sepsis that led us to suppose that another cause might be responsible for this lesion. The postoperative course was uneventful, and no symptoms of intestinal ischemia were noted.

Worldwide, there have been only eleven reports of IMA aneurysms associated with tight stenosis of the SMA and celiac trunk [5,6]. Other publications have presented cases with abdominal angina and weight loss [7] or even rupture [6]. Previous authors have conjectured that this situation may arise because of a ''jet disorder'' phenomenon. Concurrent occlusion of the superior mesenteric and celiac arteries leads to greatly increased and possibly turbulent blood flow in the IMA [8]. This produces localized areas with high arterial pressure that in an unadapted vessel can lead to aneurysm formation and subsequently thus to rupture, as was the case in our patient. The marginal artery of Drummond was also greatly increased in size (Fig. 2) and supplied the entire gastrointestinal tract. Today, abdominal ultrasound, computed tomography, magnetic resonance imaging, and arteriography facilitate an early diagnosis. Although these patients are generally asymptomatic, it is generally acknowledged in literature, to treat VAA because of the risk of rupture or ischemia. Either surgical or endovascular therapeutic procedures can be performed in the treatment of this lesion and are well described in literature [4]. Percutaneous transcatheter coil embolization techniques are also used with increasing frequency in the treatment of VAA [9]. Nevertheless, a high incidence of aneurysmatic sac reperfusion and a relatively high morbidity are associated with this procedure. Other percutaneous techniques such as permanent liquid embolic material and the use of a covered stent have been proposed, but the results must yet be verified. In our case, because of the hypovolemic shock an emergency surgical approach was indicated. The IMA being very tortuous, thus implying a difficult endovascular approach, we decided to perform a direct anastomosis between both ends of the inferior mesenteric artery instead, which seemed more feasible in our case. In the same instance we could take advantage of this type of approach to also evacuate the symptomatic retroperitoneal hematoma mentioned above. The mesenteric circulation being not compromised, revascularisation of the SMA and celiac trunk was not undertaken.

## Conclusions

Even in cases of asymptomatic VAA, surgical repair can be performed with low morbidity and mortality rates; an aggressive surgical approach is preferred to eliminate risk of rupture or ischemia. Aneurysms of the IMA are discovered only infrequently. However, there are increasing reports of IMA aneurysms in association with occlusion of the superior mesenteric and celiac arteries. Resection, with or without reconstruction, is the method of choice for their treatment. This case report should alert gastroenterologists and surgeons to the unexpected presentation of this pathology and its multidisciplinary treatment.

## Consent

Written informed consent was obtained from the patient for publication of this case report and any accompanying images. A copy of the written consent is available for review by the Editor-in-Chief of this journal.

## List of abbreviations

VAA: visceral artery aneurysms; IMA: inferior mesenteric artery; SMA: superior mesenteric artery.

## Competing interests

The authors declare that they have no competing interests.

## Authors' contributions

PPV was the main vascular-surgeon during the operation. RCM was the colorectal surgeon responsible for the operation and who also wrote the paper. ISJ recollected the bibliography about this case. NMF was responsible for the CT-Imaging preparation. JAF reviewed the paper from the colorectal surgery point of view. VGR reviewed the paper from the vascular surgery point of view. PP was responsible for the final corrections and comments. All authors read and approved the final manuscript.

## Pre-publication history

The pre-publication history for this paper can be accessed here:

http://www.biomedcentral.com/1471-230X/10/59/prepub

## References

[B1] García de la Torre A Lozano P Corominas C Juliá J Blanes I Flores D Rimbau E An aneurysm of the inferior mesenteric artery associated with obstruction of the superior mesenteric artery and the celiac trunk Rev Esp Enferm Dig 1995 87 255 8 7742056

[B2] Davidovic LB Vasic DM Colic MI Inferior mesenteric artery aneurysm: case report and review of the literature Asian J Surg 2003 26 176 9 10.1016/S1015-9584(09)60379-1 12925294

[B3] Carr SC Mahvi DM Hoch JR Archer CW Turnipseed WD Visceral artery aneurysm rupture J Vasc Surg 2001 33 806 811 10.1067/mva.2001.112320 11296336

[B4] Sacar M Tulukoglu E Ucak A Guler A Turan Yilmaz A Inferior mesenteric artery aneurysm combined with renal artery stenosis in a patient with neurofibromatosis Perspec Vasc Surg Endovasc Ther 2006 18 3 217 20 10.1177/1531003506295101 17172535

[B5] Mandeville KL Bicknell C Narula S Renton S Inferior mesenteric artery aneurysm with occlusion of the superior mesenteric artery, coeliac trunk and right renal artery Eur J Vasc Endovasc Surg 2008 35 312 313 10.1016/j.ejvs.2007.07.018 17913522

[B6] Raju RS Surnedi MK Sitaram V Govil S Inferior mesenteric artery aneurysm: case report and literature review Trop Gastroenterol 2005 26 3 139 40 16512464

[B7] Araji O Barquero JM Marcos F Infantes C Inferior mesenteric artery aneurysm associated with occlusion of the superior mesenteric and celiac arteries Ann Vasc Surg 2001 15 399 401 10.1007/s100160010073 11414095

[B8] Sugrue ME Mehigan D Hederman WP Inferior mesenteric artery aneurysm J Cardiovas Surg 1990 31 380 381 2370273

[B9] Chiesa R Astore D Guzzo G Frigerio S Tshomba Y Castellano R de Moura MR Melissano G Visceral artery aneurysms Ann Vasc Surg 2005 19 1 42 8 10.1007/s10016-004-0150-2 15714366

